# The Prevalence of High-Risk Human Papillomavirus in Hungary—A Geographically Representative, Cross-Sectional Study

**DOI:** 10.3389/pore.2022.1610424

**Published:** 2022-06-15

**Authors:** András István Fogarasi, Márta Benczik, Ágota Moravcsik-Kornyicki, Adrienn Kocsis, Anikó Gyulai, Zsigmond Kósa

**Affiliations:** ^1^ SYNLAB Genoid Molecular Diagnostic Laboratory, SYNLAB Hungary Ltd., Budapest, Hungary; ^2^ Department of Health Visitors Methodology and Prevention, University of Debrecen, Nyíregyháza, Hungary; ^3^ Department of Preventive Health Sciences, Institute of Applied Health Sciences, University of Miskolc, Miskolc, Hungary

**Keywords:** cervical cancer, cancer screening, cross-sectional studies, genotype, human papillomavirus infection, human papillomavirus vaccines, prevalence studies, rural population

## Abstract

**Background:** The estimated age-standardized incidence and mortality rates of cervical cancer in Hungary are substantially higher than the European average. In many countries, human papillomavirus (HPV) testing is the first-line method of cervical cancer screening in women >30 years. According to the European guidelines, evidence-based improvement of a national prevention strategy requires the monitoring of representative data.

**Methods:** ThinPrep cervical samples were collected over a period of 8 months at 84 sampling sites, including 4,000 eligible samples with valid laboratory results from the screening target population of females aged 25–65 years, with addresses in the representative geographic area (19 counties and four major settlement types). Genotyping of high-risk HPV (hrHPV) was performed using the Confidence HPV-X (Neumann Diagnostics) and Linear Array HPV Genotyping (Roche) tests. Demographic data were collected using a questionnaire, enabling the analysis of hrHPV genotype distribution by age, geography, education, and HPV vaccination.

**Results:** Overall, 446 samples were hrHPV-positive, showing a prevalence of 11.15% (9.73% age-representative), similar to the world average, higher than the European average, and lower than the Eastern-European average. After age standardization, no significant geographic differences were found, except for low hrHPV prevalence in villages (*p* = 0.036) and in those with elementary education (*p* = 0.013). Following genotypes 16 and 31, in order of frequency, certain non-vaccine hrHPV genotypes (HPV51, 66, 56) showed unexpectedly higher prevalence than international data.

**Conclusion:** Our study provides the first geographically representative genotype-specific hrHPV prevalence baseline database in Hungary to support policy-making efforts. Significant correlations with demographic data have transferable conclusions.

## Introduction

The estimated age-standardized incidence and mortality rates of cervical cancer in Hungary (17.2 and 4.9 per 100,000 women, respectively) are substantially higher than the European averages (10.7 and 3.8 per 100,000 women, respectively). In regional comparisons, Central and Eastern Europe had notably higher incidence and mortality rates (14.5 and 6.1 per 100,000 women, respectively) than Western, Southern, and Northern Europe (7.0, 7.7, and 10.4 per 100,000 women and 2.0, 2.3, and 2.2 per 100,000 women, respectively). In addition, the age-standardized overall cancer mortality in Hungary was found to be the least favorable in the European Union, adding a burden to an ageing population, economy, and health care system (1).

Testing for high-risk genotypes of human papillomavirus (hrHPV) has already been widely considered a more effective first-line screening method for preventing cervical cancer than the conventional Papanicolaou Pap-smear. This also requires less frequent screening intervals, owing to its superior sensitivity and negative predictive value (2). The European Guideline has recommended the use of hrHPV screening alone for women aged >30 years (3). Therefore, many countries have implemented this in their public health policies and practices.

In addition to optimizing immunization, screening, triage, and therapy procedures, evidence-based improvement of national prevention policy requires the collection and evaluation of representative data to continuously monitor the efficacy of changes, better understand and respond to demographic differences, and focus efforts and resources accordingly. Our study aimed to provide the first geographically representative baseline hrHPV genotype database for Hungary to prepare an update in the guidelines for HPV-based screening, shortly after the introduction of the nonavalent vaccine, replacing the bivalent one, into the nationwide school vaccination program.

## Materials and Methods

Our objective was to collect and analyze 4,000 cervical samples from women aged 25–65 years who were inhabitants of settlements geographically representative of Hungary’s such sub-population. A predetermined patient number matrix was prepared for each of Hungary’s 19 counties and stratified according to the four major settlement type levels (capital, county-level city, town, and village). This reflected the previously published official demographic data on women aged 25–65 years (see grey part of Table 1).

**TABLE 1:** Number of samples (N^o^) from and hrHPV prevalence in the different counties and settlement types of Hungary.

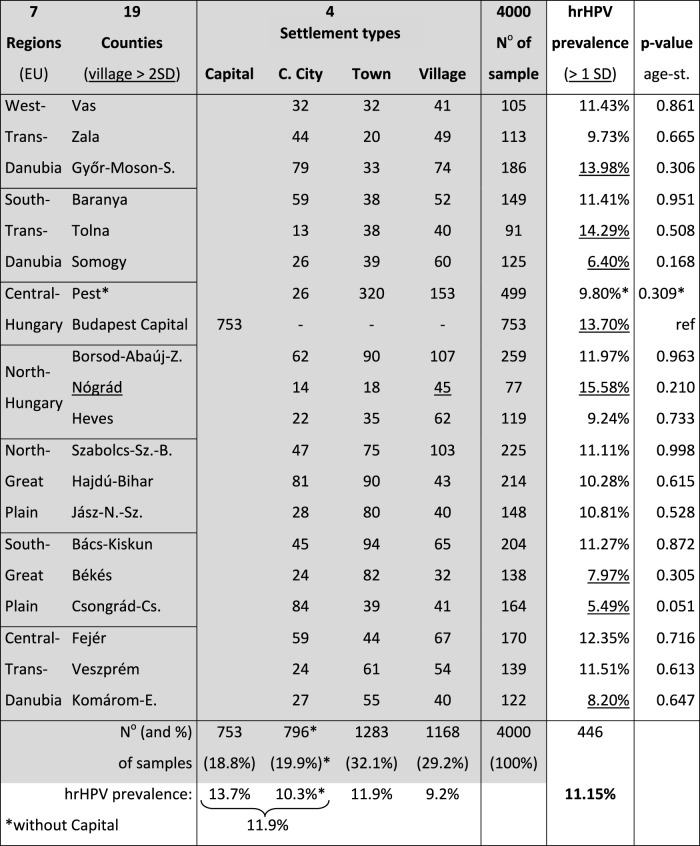

Remarks: County with rate of rural village population outside two standard deviations from country average without capital is underlined. hrHPV prevalence values outside one standard deviation from the country average are underlined. hrHPV prevalence in Pest county including Capital was 12.14%. C. City, County-level city. age-st., age-standardized to the country.

A total of 4,731 cervical samples were collected using the Rovers Cervex-Brush Combi RT™ device (Rovers Medical Devices; Oss, Netherlands) into ThinPrep PreservCyt™ liquid-based cytology containers (Cytyc Corporation; MA, United States) over a period of 8 months (December 2018–July 2019) by 84 competent sampling sites that were appointed randomly. These included 4,000 eligible samples with valid laboratory results from the screening target population of females aged 25–65 years, with addresses in the representative geographic area (counties and settlement types). We excluded participants with histories of hysterectomies, immunosuppression, recent (within 2 months) childbirths, operations or samplings that affected the cervix, and current menstruation. In each county, there were an average of 2–3 health care institutions and 2–3 registered health visitors who collected samples from women after securing their informed consent, guided by the approved standard recruitment, sampling, and documentation protocol in compliance with the Declaration of Helsinki (ethical approval obtained from ETT TUKEB; approval no. 61407-2/2016/EKU).

The detection and genotyping of hrHPV were performed through nucleic acid testing (multiplex real-time PCR amplification) using the Confidence HPV-X™ (Neumann Diagnostics; Budapest, Hungary) and Linear Array™ HPV Genotyping (Roche; Basel, Switzerland), which are both clinically validated commercial CE-marked *in vitro* diagnostic tests (4). The laboratory that performed the tests was selected through a public procurement process. The procedure commenced with the primary HPV test, using the QuantStudio™ 6 Flex (LifeTechnologies; CA, United States) platform, to detect all 14 hrHPV genotypes. However, only seven hrHPV genotypes were detected individually (HPV 16, 18, 31, 33, 45, 52, 58, present in the 9-valent vaccine), while the rest were detected in a group (HPV 35, 39, 51, 56, 59, 66, 68—66 and 68 have been re-classified by the World Health Organization as “potentially hrHPV”). Non-template preparation and PCR control, human control, internal control, PCR positive control, and contamination cut-off were applied for technical validation, resulting in 2.2% invalid results in the first test. When an hrHPV genotype was detected in the group, a secondary HPV test using the Linear Array™ HPV Genotyping test was conducted for individual identification.

A logical decision-making algorithm was designed to settle possible inter-test inconsistencies through single repetition of one or each of the tests for consistency or exclusion. The rate of invalid results for the second test, including repeated inconsistencies, was 1.5%. We utilized two consecutive HPV tests for cost-effectiveness; the first test saved costs by group detection in negative samples and only nonavalent genotype positives, so the second genotyping test was needed for a smaller fraction (303/4,731, 6.4%) of the whole sample set.

An anonymous questionnaire collecting demographic (city of permanent address, birth date, and highest level of education) and HPV vaccination data was given to each patient, allowing us to understand hrHPV genotype distribution and perform further cross-analyses using subgroups according to geography, age, education, and vaccination.

A descriptive statistical analysis was used to compute the frequency rates of each variable. Differences between the counties and the national averages were analyzed and statistically (95% MT; *p* ≤ 0.05) demonstrated using two-sample t-tests with STATA software (version 13.0; TX, United States). In addition, the relationship between variables was analyzed using analysis of variance and logistic regression models to estimate the odds ratios.

## Results

The overall prevalence of hrHPV was 11.15% (446 high-risk HPV-positive samples out of 4,000; 95% confidence interval [CI]: 10.05–12.25%) before age standardization. After adjusting for age to better represent female Hungarians aged 25–65 years, it decreased to 9.73% (95% CI: 8.31–11.15).

### Patients

The mean age of the 4,000 eligible women was 41.36 years (standard deviation [SD]: 9.972). Of the eligible samples, 31.3% were from the Capital and Central Region, 30.0% from the three western regions, and 38.7% from the three eastern regions, proportional to the population. In total, 29.2% of the population lived in villages. However, there was a wide variation in this proportion among counties (SD = 10.0). It was beyond 2 SD in one of the 19 counties, significantly above country average without capital (Nógrád county, *p* = 0.028, underlined in Table 1).

### Geographic Distribution

**FIGURE 1:**
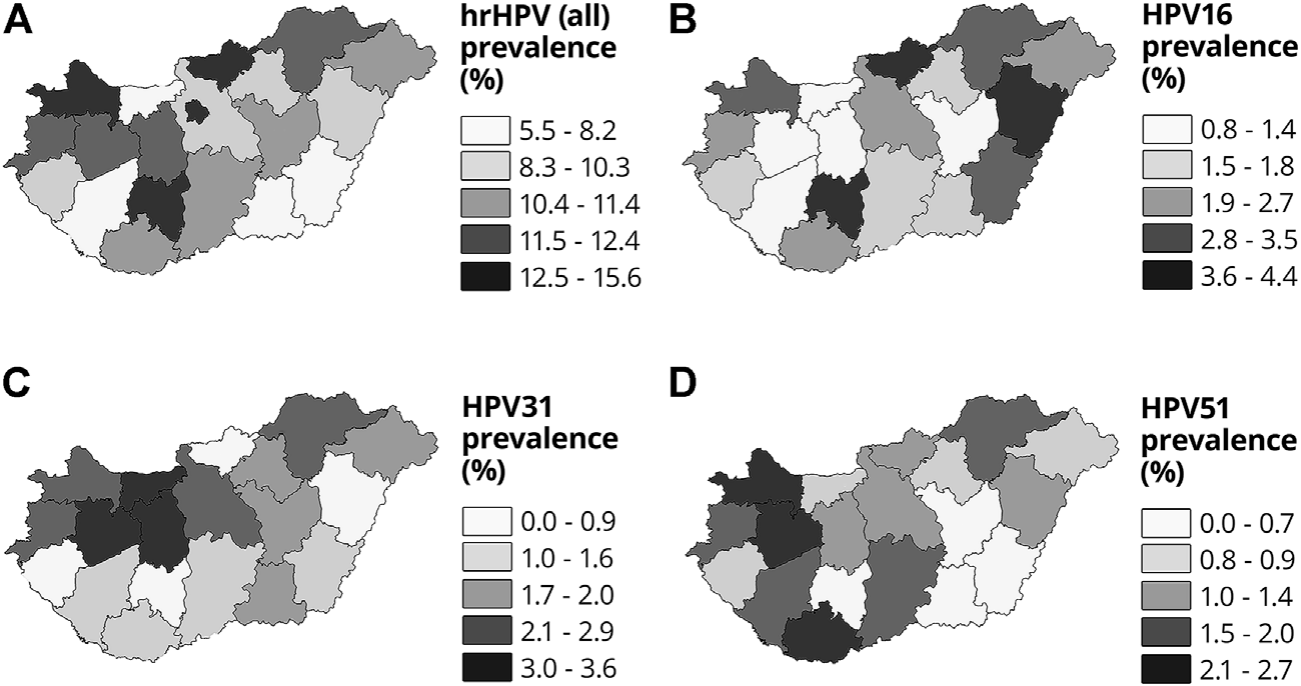
Geographic representation of the prevalence of **(A)** all hrHPV, **(B)** HPV16, **(C)** HPV31, **(D)** HPV51 in the counties of Hungary.

Table 1 summarizes the total samples and the rate of hrHPV-positive samples in each county and by settlement type. Figure 1A presents graphical representation of hrHPV prevalence in each county (color depth-coded ranges). Without age standardization, hrHPV prevalence was beyond 1 SD higher in the Nógrád, Tolna and Győr-Moson-Sopron counties and in the Capital and beyond 1 SD lower in the Komárom-Esztergom, Békés, Somogy and Csongrád-Csanád counties than the country average.

### Education

A higher level of education was associated with a higher (almost double) prevalence of hrHPV: hrHPV prevalence among women with “High school or University degrees” was 12.8% (*p*-value as reference), while among those with “Less than Elementary” education 6.7%. hrHPV prevalences among women with “Middle education level (College, Matura, 12 years of education),” with “Higher than Elementary education (but without Matura),” and with just “Elementary level (8 years of education)” with OR and *p*-values are presented in Table 2.

**TABLE 2:** hrHPV prevalence by groups with the highest education level.

Highest level of education	hrHPV prevalence	OR	p-value
High school or University degree	12.8 %	ref	ref
Middle (College, Matura, 12 years of education)	10.5 %	0.843	0.159
Higher than Elementary (without Matura)	12.1 %	1.058	0.720
Elementary (8 years of education)	7.2 %	0.550	0.013^∗^
Less than Elementary	6.7 %	0.477	0.481

a Significant *p* ≤ 0.05.

A significant difference (*p* ≤ 0.05) was found only between “Elementary level (8 years of education)” versus “High school or University degrees” (OR = 0.550, *p* = 0.013), even without the effect of age. Meanwhile, the size of the “Less than Elementary” subgroup was too small (*n* = 15) to be significant.

### Age Correspondence

**FIGURE 2:**
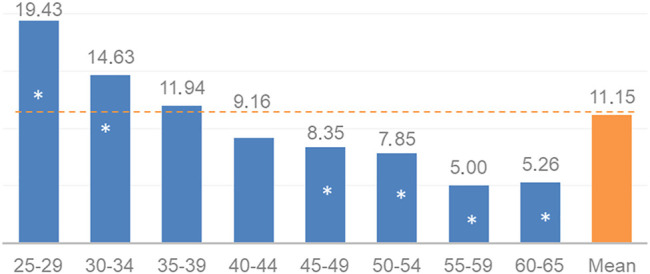
Prevalence of hrHPV (%) by 5-year age groups among Hungarian women aged 25–65 years (significant differences from average at level *p* ≤ 0.05 are marked*).

The prevalence decreased by age, from 19.43% in the 25–29 years age group, through 14.63% in the 30–34 age group, 11.94% in the 35–39 age group, 9.16% in the 40–44 age group, 8.35% in the 45–49 age group, and 7.85% in the 50–54 age group, to 5.0% and 5.3% in the 55–59 and 60+ years age groups, respectively. Figure 2 shows hrHPV positivity (%) in 5-year age groups from 25 to 65 years on a graph.

**FIGURE 3:**
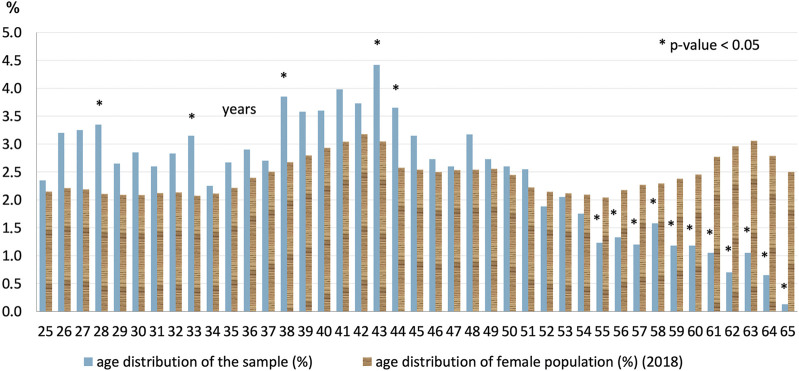
Age distribution (%) of the sample set (*n* = 4,000) and the female population aged 25–65 years in Hungary (2018) (*significant differences at level *p* ≤ 0.05). Age in completed years.

During our sample collection, the representativeness to the age distribution of the female population was not a requirement. However, we ensured the validity of the conclusions for the country through statistical adjustments. Analysis of the demographic data in the sample set showed that women under the age of 51 were overrepresented, while those aged 52 years and above were underrepresented (see Figure 3). After eliminating the effect of mean patient age differences by aligning the size of each age subset in our model (see *Discussion - Age Correspondence*), the hrHPV prevalence representative of the female Hungarian population aged 25–65 years was determined to be 9.73%.

### Vaccination

Based on our data, 174 enrolled women (4.3%) had received vaccination against HPV; among them, 55 (32%) received the quadrivalent vaccine; 47 (27%), the bivalent; 16 (9%), the latest nonavalent vaccine; while 56 (32%) did not know the type. The average age at the time of the survey was 36 years and that at the time of vaccination was 27 years. Among the reported vaccinated women, 11 were hrHPV-positive, and four among them had a genotype matching her previous vaccine. The prevalence of HPV16 and/or HPV18 was 10% among the vaccinated vs. 29% among the unvaccinated hrHPV-positive women (*p* = 0.211).

### Genotypes

**FIGURE 4:**
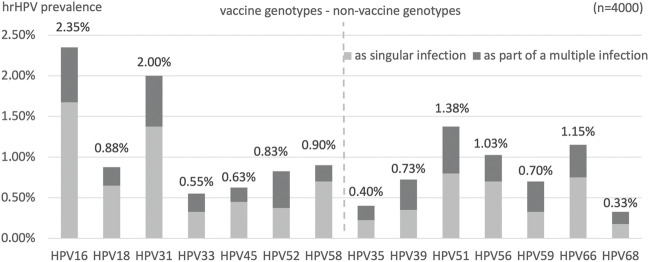
Type-specific prevalence (%) of each hrHPV genotypes. Divided if singular infection or as part of a multiple hrHPV infection.

Among the 446 positive samples, a single hrHPV was detected in 355 (79.6%) samples. Multiple infections were present in 91 cases (20.4%); with 76 (17.0%) having a double infection, 13 (2.9%) having a triple infection, and two (0.5%) having a quadruple infection with different hrHPV genotypes. The frequencies of the examined hrHPV genotypes are shown in Figure 4, divided if singular or as part of a multiple infection.

Immediately following genotypes 16 and 31 (together comprising 39% of positives) in order of frequency, certain non-vaccine high-risk HPV genotypes (HPV51, HPV66, HPV56) had the highest prevalence (together comprising 32% of positive cases).

**FIGURE 5:**
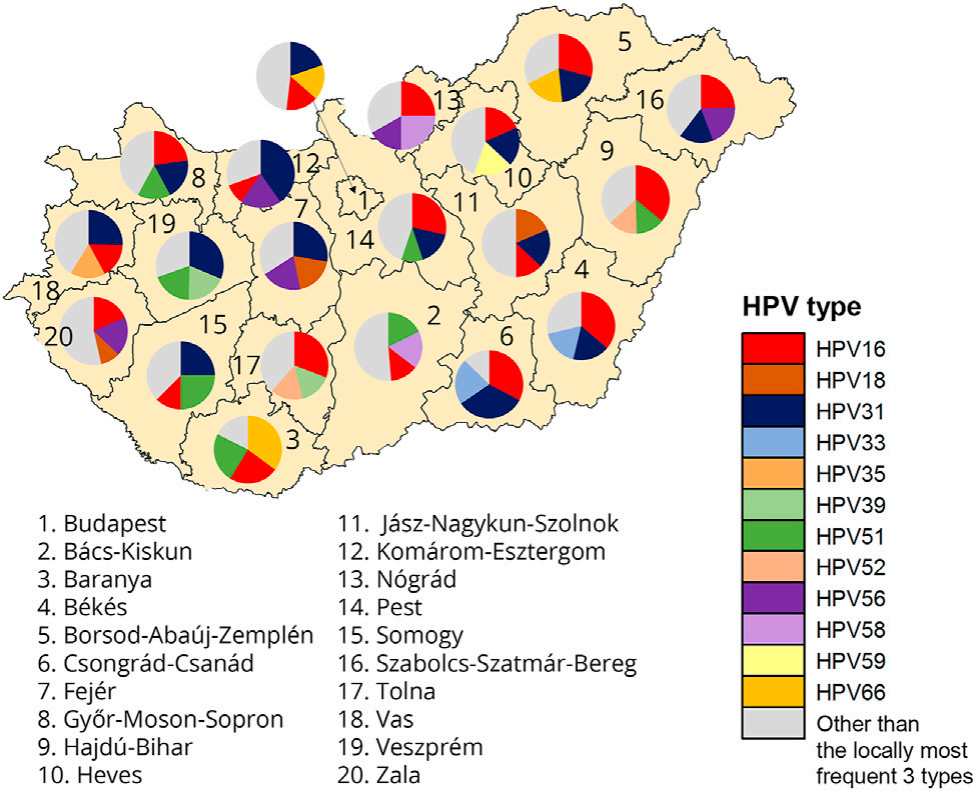
Geographic representation of rate of the three most frequent genotypes per county among Hungarian women aged 25–65 years.

The geographic distributions of HPV 16, 31, and 51 are shown in Figures 1B–D, respectively, for comparison. HPV66 was mainly present in the capital and Baranya county. Figure 5 shows the rate of the three most prevalent genotypes per county.

Bivalent vaccine-types (HPV16 and/or HPV18) were present in 110 females (29%), while any or more of the nonavalent vaccine 7 hrHPV genotypes were found in 269 (73%) of the positive samples.

**FIGURE 6:**
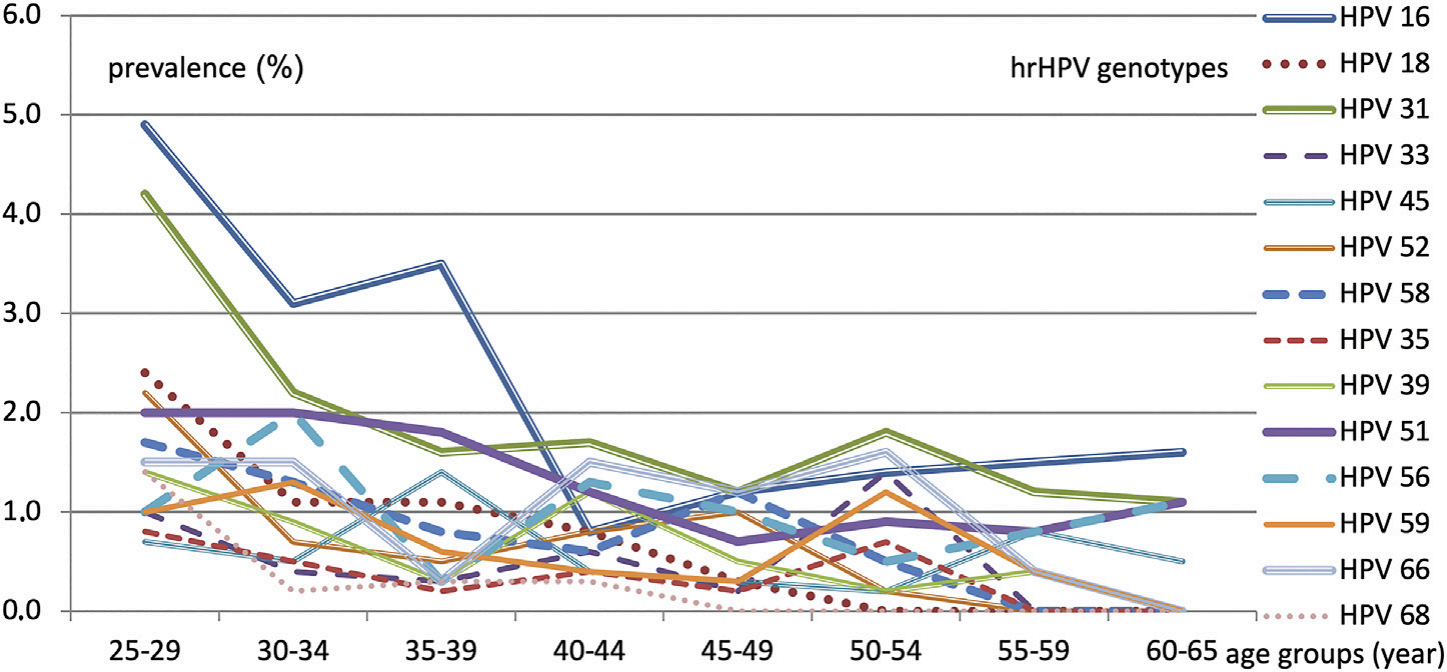
hrHPV genotype prevalence (%) according to each 5-year age group.

As shown in Figure 6, the prevalence of individual hrHPV genotypes decreased with age, though some had a minor second increase. The most pathogenic HPV16 had the highest prevalence at 4.9% in the 25–29 years age group, which remained above 3% in the 30–34 and 35–39 years age groups but decreased to approximately 1% in the 40+ years groups. In the 25–29 years age group, the following other genotypes had prevalence above 2%, in their order of frequency: HPV 31, 18, 52, 51. Approximately 2% of the patients tested positive for HPV31, 51, and 56 in the 30–34 years age group.

## Discussion

The nationwide prevalence of 11.15% was similar to the world average of 11.7%, based on normal cytology, lower than that of the Eastern-Europe average (21.4%) (5), but higher than that of most Western European countries, ranging from 1.7% to 12.5% (6). Some Western European countries have a higher hrHPV prevalence (presumably migration-related) but lower morbidity and mortality rates of cervical cancer than Hungary, providing a benchmark for improving the effectiveness of secondary and tertiary prevention.

### Previous Studies

In Central and Eastern Europe, the mean prevalence of HPV infection was 12.6% among 8,610 women with normal cervical cytology, with HPV16 being the most common (7). Some countries in this subregion have already published more detailed population-based screening data on hrHPV prevalence with substantial variance (8, 9).

In Hungary, only a few studies have been previously conducted that covered HPV prevalence. One study in 2001 examined 728 women in more centers and estimated that the overall rate of HPV infection was 17% (10). Another study in 2002 collecting 1,121 samples from two major cities found a 17.5% HPV prevalence in a healthy population with broad regional (15%–27%) and age group (10%–32%) differences. At the Szeged center, the HPV-positivity was 27.6%, whereas at the three centers in Budapest, HPV prevalence did not exceed 15% (11). In 2006, one of the authors of this paper found a genotype frequency rank of 16, 31, 51, 66, 56, 58, 33, 39, and 18 in 75% monovalent, 20% bivalent, and 5% infections with three or more genotypes (12). A single HPV-center (University Clinic) examined 1,155 women (not screening population) and found that 55.5% of patients tested positive for HPV DNAs, with 38.5% having high-risk HPV DNA. The most common HPV type found was type 16 (19.5%), with high prevalence of type 51 and 31 among patients with cytological atypia (13). A study in 2016 examined the HPV genotypes from 2048 cytology results (not in a screening population) and found an hrHPV positivity of 12.7%, with the most prevalent (ranked in order) being 16-52-51-31-66-58 (14).

However, none of these earlier studies attempted to proportionally represent the urban and rural populations of the whole country, making our recent study the first and unique survey in this aspect.

### Geographic Distribution

The distribution of local hrHPV prevalence can be compared to the strong geographic inhomogeneity in the morbidity and mortality of cervical cancer (15). In some counties, the relatively high hrHPV prevalence correlated with the higher morbidity and mortality rates (e.g., Nógrád, despite the high >2 SD rural population rate) or lower prevalence with lower morbidity and mortality (e.g., Komárom-Esztergom). However, in other counties, only the morbidity rate matched but mortality did not (high prevalence and high morbidity, e.g., Tolna; low prevalence and low morbidity, e.g., Somogy). In contrast, in some counties, these did not correlate at all (high prevalence with low morbidity and mortality, e.g., Győr-Moson-Sopron, and low prevalence with high morbidity and mortality, e.g., Békés, Csongrád-Csanád). This is explained by the indirect chain of relation between prevalence and morbidity, highly influenced by the genotype pathogenicity and effectiveness of screening and therapy efforts.

It is important to consider the potentially significant effect of the different settlement types and age distribution among the counties to better understand the observed hrHPV prevalence differences. In urban areas (towns and county-level cities, with the highest prevalence in the capital), the prevalence was 11.9%, while in villages, it was significantly (*p* = 0.036) lower (by 23%) at 9.2%. This finding was not unprecedented as a similar difference was found in Romania (9), where hrHPV prevalence was 20.0% and 14.8% (lower by 26%) in urban and rural areas, respectively. However, in Romania, the logistic regression model did not show a significant association between hrHPV positivity and settlement type, while it did in Hungary.

### Education

Although the results of our study suggest that hrHPV prevalence is higher in urban population groups with high school or university degrees, we know from other study results that this may be an effect of HPV screening compliance as well. Higher level of HPV knowledge was significantly associated with HPV testing behavior (odds ratio: 3.792, 95% CI: 3.400–4.230). The effect of residence and educational attainment on testing behavior only became significant if women had low levels of HPV knowledge (16).

### Age Correspondence

Similar to previous findings (17), the hrHPV positivity rate had a strong negative correlation with age until a certain middle age group, when the graph plateaued or only increased slightly. The prevalence in females in their 20s was approximately 20%, falling to <10% among those in their 40s and approximately 5% among those around the age of 60, suggesting an exponential function. In our applied regression model (negative log), the change in the odds ratio of hrHPV positivity with each year of aging (additionaly) did not differ significantly (*p* = 0.726) from 4% per year (mathematically multiplied by 0.96^y^) for the entire age range. The use of this simplified model can achieve the aim of filtering out the significant (regression analysis *R* ^2^ = 0.0016, *p* < 0.05) effect of age.

Based on the literature, the peak of HPV prevalence is in the early 20s, which presumably correlates with the initiation of sexual activity and the number of different partners over a certain period of time. Cervical pre-cancer findings cumulate approximately 10 years after this age, while the peak of cervical cancer appears even later. Only approximately 10% of HPV infections persist over 2 years, and persistence with the same hrHPV genotype indicates a higher risk for cervical neoplasia (18).

As mentioned before, analysis of the demographic data in our sample set showed that women under the age of 51 were overrepresented, while those aged 52 years and above were underrepresented (see Figure 3), which reflects also an important aspect of age differences in the attendance of and compliance with cervical screening programs. Adjustment to the 2000–2025 World Health Organization World Standard Population might permit better international comparison, with the limitation that standard age distribution data combine the data for males and females.

Among the different counties of Hungary, differences in the hrHPV prevalence from the country average (see Table 1) were not significant (*p* = 0.243) without the effect of age differences, suggesting that domestic screening programs should be targeted based on age groups instead of geographic regions. However, the hrHPV prevalence remained significantly lower in village settlement types than in the capital, even after adjusting for age.

### Vaccination

The average age at the time of reported vaccination was 27 years in our study group, suggesting that most of the patients received vaccination after engaging in sexual activity. In their cases, there may be incomplete protection because of the potential presence of hrHPV genotypes that were already in the cervix. Indeed, among the reported vaccinated women, 11 were hrHPV-positive, and four of them had a genotype in her cervix against which she received lege artis vaccination in the previous years (HPV16 — vaccination age 34 — quadrivalent; HPV16,58 — vaccination age 45 — nonavalent; HPV18 — vaccination age 28 — nonavalent; HPV31 — vaccination age 36 — nonavalent).

Vaccination can be also an important aspect in achieving a higher level of knowledge, according to a previous research result, which found that vaccinated women were more likely to know that screening should be continued despite vaccination (60.0% vs. 25.6%, *p* = 0.06) (19).

### Genotypes

Individual genotyping of each positive sample was included in our study design because the risk of HPV-related pre-cancer differs substantially among the individual hrHPV genotypes (20). Specifically, in a prospective study (*n* = 8,656, follow-up 12 years), when HPV16 was present in the cervix, the risk of CIN3+ (cervical intraepithelial neoplasm grade 3 or worse) was more than 25%, with HPV18 it was approximately 20%, with HPV31 and HPV33 approximately 15%, and with the rest of the hrHPV genotypes only <5% (18).

Immediately following genotypes 16 and 31, in order of frequency, certain non-vaccine high-risk HPV genotypes (HPV51, 66, 56) had unexpectedly high prevalence in Hungary compared with international data. The latter three were not listed in the five most frequent worldwide, a surprising result that necessitates further research to be understood.

Worldwide, the following genotypes were the most prevalent: HPV16 (3.2%), HPV18 (1.4%), HPV52 (0.9%), HPV31 (0.8%), HPV58 (0.7%), and the remaining hrHPV are 0.6% or less (21). Another high-population study found that HPV16 (1.6%) and HPV52 (1%) were the most prevalent in the United States (22). Meanwhile, HPV16 and HPV31 were the two most prevalent in Europe (21% and 9% among positives, respectively) in contrast with other continents where other genotypes came up earlier in the frequency rank list: Asia: HPV33 (6%), HPV56 (6%), South-America: HPV58 (7%), and Sub-Saharan Africa: HPV35 (8%) (23).

In our geographically-representative screening population, the most frequent genotypes were similar to those found in some of the earlier studies in Hungary. Genotype 16, 51 and 31 had the highest prevalence in 2011 among patients with cytological atypia (13). A study in 2016 examined the HPV genotypes from 2048 cytology results (not in a screening population) the most prevalent (ranked in order) being HPV16-52-51-31-66-58 (14). Five out of the six most frequent genotypes were similar to our findings, with less HPV56 and more HPV52. HPV51 deserves special attention according to all genotyping studies in Hungary.

### Summary/Conclusion

Key Points: According to Our Results

• A hrHPV prevalence of 11.15% (9.73% age-representatively) was found in Hungary, similar to the world average, higher than the European average, and lower than the Eastern-European average;
• Domestic screening programs should focus on age group rather than geographic regions, with an attention to the involvement of women above 51;
• High education level and urban residence (even after age-standardization) was associated with statistically significantly higher hrHPV prevalence;
• Following genotypes 16 and 31, in order of frequency, certain non-vaccine hrHPV genotypes (HPV51, HPV66, HPV56) showed unexpectedly higher prevalence than previous international data, projecting a potential tendency of further growing relative prevalence of these non-vaccine genotypes.

Evidence supports the introduction of primary HPV screening in Hungary. Our study provided the first geographically representative genotype-specific hrHPV prevalence baseline database for supporting policy-making efforts. Further follow-ups should reveal probable genotype distribution shifts over time toward the growing prevalence of the non-vaccine genotypes, in conjunction with HPV vaccination and organized cervical screening programs.

## Data Availability

The raw data supporting the conclusion of this article will be made available by the authors, without undue reservation.
